# Effect of Neem Leaf Extract (*Azadirachta indica*) on c-Myc Oncogene Expression in 4T1 Breast Cancer Cells of BALB/c Mice

**Published:** 2012-06-13

**Authors:** Fauziah Othman, Gholamreza Motalleb, Sally Lam Tsuey Peng, Asmah Rahmat, Rusliza Basri, Chong Pei Pei

**Affiliations:** 1. Department of Human Anatomy, Faculty of Medicine and Health Sciences, University Putra Malaysia, Selangor, Malaysia; 2. Department of Biology, Faculty of Science, University of Zabol, Zabol, Iran; 3. Department of Nutrition and Dietetic, Faculty of Medicine and Health Sciences, University Putra Malaysia, Selangor, Malaysia; 4. Department of Biomedical Science, Faculty of Medicine and Health Sciences, University Putra Malaysia, Selangor, Malaysia

**Keywords:** Breast Cancer, c-Myc Gene, Primed *in situ* Labelling

## Abstract

**Objective:**

Breast cancer is the most common cause of cancer-related deaths in women both worldwide and in Malaysia. *Azadirachta indica* (A. Juss), commonly known as neem, is one of the most versatile medicinal plants that has gained worldwide prominence due to its medicinal properties. However, the anticancer effect of ethanolic neem leaf extract against breast cancer has not been documented. The purpose of the present study is to investigate the effect of neem leaf extract on c-Myc oncogene expression in 4T1 breast cancer BALB/c mice.

**Materials and Methods:**

In this experimental study, A total of 48 female BALB/c mice were divided randomly into four groups of 12 mice per group: i.cancer control (CC) treated with 0.5% Tween 20 in PBS, ii. 0.5 µg/mL tamoxifen citrate (CT), iii. 250 mg/kg neem leaf extract (C250), and iv. 500 mg/kg neem leaf extract (C500). *in situ* reverse transcription polymerase chain reaction (*in situ* RT-PCR) was applied to evaluate suppression of c-Myc oncogene expression in breast cancer tissue.

**Results:**

The C500 group showed significant (p<0.05) suppression of c-Myc oncogene expression compared to the CC group.

**Conclusion:**

c-Myc was found to be down regulated under the effect of 500 mg/kg ethanolic neem leaf extract.

## Introduction

Breast cancer is the most common cause of cancer death in women and is a growing health problem in Malaysia. According to the 2003 National Cancer Registry Report, breast cancer was the most common cancer for all ethnic groups, among all females from the age of 20, with an age specific incidence rate of 52.8 per 100,000 (1). Despite the presence of several recognized risk factors such as early menarche, late menopause, nulliparity, and positive family history, there is no realistic option for primary prevention of breast cancer in individuals at the present time (2).

Oncogenes are gene encoding proteins involved in cell cycle regulation. They sustain numerous genetic damages and produce proteins capable of cellular transformation. Mutated or abnormally expressed oncogenes under extreme conditions convert normal cells to cancer cells. Oncogene products are found in different subcellular compartments such as plasma membrane (ras, srs, abl), cytoplasm (raf, erb-H), and nucleous (myc, fos, ski, jun). *Azadirachta indica* (A. Juss), commonly known as neem, is one of the most versatile medicinal plants that has gained worldwide prominence owing to its medicinal properties. Some of its impressive therapeutic qualities are its anti-viral, anti-microbial, anti-inflammatory, anti-tumour, anti-pyretic, anti-bacterial, anti-fungal, and anti-hyperglycemic properties (3).

Recently neem has been reported to induce apoptosis in the MCF-7 breast cancer cell line (4). Several studies have demonstrated that alcoholic extracts of neem leaf are more effective than aqueous extracts for cancer treatment (5). However, the anticancer effect of ethanolic neem leaf extract against breast cancer has not been documented. The purpose of the present study is to investigate the effect of neem leaf extract on oncogene (c-Myc) expression in 4T1 breast cancer BALB/c mice.

## Materials and Methods

### Cell culture

Mouse mammary tumour cells (4T1) were purchased from American Type Culture Collection (ATCC; cat. no. CRL2539). All culture work was performed under strict aseptic conditions. Cells were cultured in 10% RPMI-1640 (R1383, Sigma, St. Louis, Mo, USA) cell culture media supplemented with 10% fetal calf serum (FCS; Sigma Aldrich, USA) and 1% penicillin/streptomycin (Sigma Aldrich, USA) in a humidified incubator supplied with 5% CO_2_ at 37℃. The cells were counted using a haemocytometer (Hawksley, England).

### Ethanolic neem leaf extraction

Neem leaf was collected from the UPM area, Selangor, Malaysia. The leaf was identified by Mr. Tajuddin Abdul Manaf, Agriculture Assistant in the Laboratory of Natural Products, Institute of Bioscience, University Putra Malaysia. The neem leaf extract was prepared as described before (6), and left to dry naturally. The dried leaf was then ground using a fine grinder to obtain the fine powdered form. A total of 100 g of powdered neem leaf was transferred into a borosilicate glass bottle to which 200 ml of 80% ethanol was added. The mixture was mixed and kept overnight at room temperature. The next day, the mixture was filtered into a beaker while the residue was left in the borosilicate glass bottle. Another 200 ml of 80% ethanol was then poured into the borosilicate glass bottle to soak the remaining residue, which was then kept overnight at room temperature. These steps were repeated for another three consecutive days. Ethanolic extract was evaporated using a rotary evaporator (Rotavapor R-300 BUCHI, Switzerland) at 55℃. Further drying was done using a freeze system (FreeZone 77520, Labconco, USA) at -80℃ for 24 hours, after which the extract was oven-dried for an additional 48 hours and then stored at 4℃.

### Animals

Female BALB/c mice, three to four weeks old, were purchased from the Institute of Medical Research (IMR), Kuala Lumpur, Malaysia and were ethically approved by the Animal Care and Use Committee (ACUC), Faculty of Medicine and Health Sciences, University Putra Malaysia. The mice were housed six to a cage at room temperature with a 12 hour light/12 hour dark schedule and received autoclaved food and water ad *libitum*.

### Breast cancer induction

Tumour development was carried out according to the modified Xanthopoulos method (7). A total of 48 female BALB/c mice were divided randomly into four groups (one cancer control group and three cancerous groups that consisted of 12 mice per group. Mice in the cancerous groups were treated as follows: i. cancer control (CC) treated with 0.5% Tween 20 in PBS; ii. cancer treated with 0.5 µg/ml tamoxifen citrate (CT); iii. cancer treated with 250 mg/kg neem (C250); and iv.cancer treated with 500 mg/kg neem (C500). Mice treated with neem received intratumoural injections of 0.1 ml of the extract every 48 hours. The mice were injected subcutaneously with 104 4T1 cells suspended in 0.1 ml of 10% RPMI-1640 in the left breast region. The tumour was detected by palpation around the induction area. The tumour was measured every two days with a digital micro caliper (Mitutoyo, Japan) and its volume (height, length, width) was calculated according to Kotoh et al. (8). The treatment was subjected for four weeks after the tumour developed. Sample collection was performed weekly after the treatment started, for a total of four weeks. At each sample collection, three mice from each group were humanely sacrificed under anesthesia.

### In situ reverse transcription - polymerase chain reaction (RT-PCR)

Formalin-fixed paraffin-embedded tumour tissues were used for *in situ* RT-PCR. The samples were sectioned from breast tissue paraffin embedded blocks 4-5 µm in thickness with a microtome (Leica RM 2135) and placed on poly-L-lysine-coated slides. The proto-oncogene (c-Myc) was selected to study changes in gene expression during neem treatment by in situ RT-PCR. β-actin was chosen as the housekeeping gene. The gene sequences of c-Myc and β-actin were obtained from the genomic DNA sequence database, GenBank (National Center for Biotechnology Information, USA). The forward (5´-GTGGGCCGCTCTAGCCACCAA-3´) and reverse (5´-TCTTTGATGTCACGCACGATTTC-3´) primers of β-actin were obtained according to Ramos-Payán et al.(9). The forward (5´CAAGAGGCGAACACACCACGTCT-3´) and reverse (5´-CCACATACAGTCCTGGATGAT-3´) primers of c-Myc were according to Coucouvanis and Jones (10). All four sets of primer sequences were sent to Germany (Operon Biotechnologies GmbH) to be chemically synthesized.

### Isolation of genomic DNA from 4T1 cells

Genomic DNA from the 4T1 tissue culture cells were isolated using a Wizard® Genomic DNA Purification Kit (Promega, USA). Initially, 4T1 cells were cultured in 75 cm^2^ tissue culture flasks in a 5% CO_2_ incubator at 37℃ until80% confluency.

The adherent 4T1 cells were trypsinized before harvesting and then transferred to a 1.5 mL micro-centrifuge tube, where they were centrifuged at 13000 rpm for 10 seconds to create cell pellets. The supernatant was then removed and 200 µL PBS was added as a wash. Cells were centrifuged as with the previous step and the PBS removed. The cells were then vortexed vigorously and resuspended. A total of 600 µL of nuclei lysis solution was added and pipetted to lyse the cells after which 3 µL of RNase solution was added to the nuclear lysate. The sample was mixed by inverting the tube 2-5 times and then incubated for 15-30 minutes at 37℃. After cooling at room temperature for 5 minutes, 200 µL of protein precipitation solution was added and vortexed vigorously at high speed for 20 seconds, chilled on ice for 5 minutes, and then centrifuged for 4 minutes at 13000 rpm. The supernatant was carefully removed and transferred to a clean 1.5 mL micro-centrifuge tube that contained 600 µL isopropanol. The solution was gently inverted until the white thread-like strands of DNA formed a visible mass, after which it was centrifuged for 1 minute at 13000 rpm at room temperature. The DNA was visible as a small white pellet and the supernatant was carefully decanted. We added 600 µL of room temperature 70% ethanol and the tube was gently inverted several times to wash the DNA. The solution was centrifuged for 1 minute at 13000 rpm at room temperature, then the ethanol was carefully aspirated and the pellet air-dried for 30 minutes. DNA was rehydrated by the addition of 30 µL of ultrapure water, incubated overnight at 4℃ and then stored at -20℃ until further use.

### Gradient PCR

Once the PCR primers were chemically synthesized, a gradient PCR was carried out to optimize the annealing temperature of each set of primers (c-Myc and β-actin). The annealing temperature for each set of primers was optimized from 50℃ to 60℃ in order to obtain the most suitable temperature for the primers to be annealed on the target sequence. A master mix that contained 10X PCR buffer, 25 mM MgCl_2_, 2 mM dNTPs, 100 pmol/µL forward, and 100 pmol/µL reverse primers was prepared and the reaction was run in a thermocycler (Biometra® T-gradient). The PCR products for each sample were electrophoresed to determine the optimum annealing temperature. The DNA and marker were electrophoresed for 45 minutes (70 volts, 165 mA) at room temperature. The gel was visualized under ultra violet (UV) light (Alpha Imager ^TM^ 2200).

### PCR purification by gel extraction

The QIAquick Gel Extraction Kit Protocol (QIAGEN) was usesd to extract and purify DNA of 70 base pairs to 10 kb from standard or low-melt agarose gels in TAE buffer. After deriving the optimum annealing temperature of c-Myc and β-actin from the gradient PCR, the PCR products of each selected gene were re-amplified and increased to a total volume of 100 µL. The mixture was loaded into the well along with 3 µL of 0.5 mg DNA/mL molecular weight marker (Fermentas 100 base pair DNA ladder marker) in the other well. The DNA and marker were electrophoresed for 90 minutes (55 volts, 165 mA) at room temperature and then the gel slice was weighed in a colorless tube. Three volumes of QG buffer were added to one volume of gel (100 mg ~ 100 µL). The gel slice in the QG buffer was incubated at 50℃ for 10 minutes. One gel volume of isopropanol was added to the sample and mixed. This step increased the yield of DNA fragments. A QIAquick spin column was placed in a provided 2 mL collection tube. To bind the DNA, the sample was added to the QIAquick column and centrifuged for 1 minute at 13000 rpm. The flow-through was then discarded and the QIAquick column was placed in the tube. Next, 0.5 mL of QG buffer was added to the QIAquick column and centrifuged for 1 minute at 13000 rpm. 0.75 mL of buffer PE was added to QIAquick column to wash and centrifuge for 1 minute at 13000 rpm. The QIAquick column was then placed into a clean 1.5 mL micro-centrifuge tube. Next, 30 µL of ultrapure water was added to the center of the QIAquick membrane to elude DNA and left for 1 minute, after which the column was centrifuged for 1 minute at 13000 rpm. The purified DNA was stored at –20℃ until further use.

### DNA sequence analysis

For DNA sequencing, we sent 70 ng/µL of purified PCR product and 20 pmoles/µL of forward and reverse primers of each of the selected genes to a company (First Base S.D.N. B.H.D, Malaysia). The BigDye® Terminator v3.1 Cycle Sequencing Kit was used to sequence DNA, with an ABI PRISM® 377 DNA Sequencer. BLAST software was used to compare the sequences of c-Myc and β-actin to the sequences in the Gene Bank.

### One-step in situ RT-PCR

Breast tumour tissue was used for in situ RT-PCR. Briefly, the tissue was washed in PBS, fixed in 10% neutral buffered formalin for at least 24 hours and then processed in an ascending series of alcohol solutions for 22 hours in an automatic tissue processor (Leica, ASP300). The tissues were then embedded into paraffin blocks with an embedder machine (Leica EG1160, Germany) and cooled at a low temperature. The tissues were trimmed and cut by microtome (Leica RM 2135, Germany) at 4-5 µm dimensions and then pasted on poly-L-lysine-coated slides (Menzel Glaser, Germany) for *in situ* RT-PCR. A modified one step in situ RT-PCR approach was performed according to Nuovo et al. (11). The slides were incubated for Two hours at 70℃ and subjected to paraffin wax removal with xylene (I, II) and decreasing concentrations of ethanol, then treated with 100 µl proteinase K (9 µg/ml; Bioron GmbH, Germany) for 10-20 minutes at 37℃. PBS and DEPC-treated water were used to wash off the proteinase K; its digestion was inactivated by incubating the slide for 1 minute at 95℃ on a heat block (Leica HI 1220). Next, slides were rinsed first in PBS and subsequently in diethyl pyrocarbonate- (DEPC) treated water, then air dried. The slides were incubated with 50 µl RNase-free DNase I (20 U) overnight at 37℃ in a humid chamber, then washed for 1 minute with DEPC water and 100% ethanol and air dried. *in situ* synthesis and amplification of cDNA were performed in one step using the QIAGEN® OneStep RT-PCR Kit (QIAGEN Hamburg GmbH, Germany) and Mastercycler® gradient PCR machine (Eppendorf, Germany). All reagents were prepared with RNase-free water. We made the glassware RNase-free by double autoclaving them at 120℃ for 20 minutes. A master mix for four reactions that contained four volumes of RNAse-free water, 5X QIAGEN OneStep RT-PCR buffer, 2 mM dNTP mix, 100 pmol/µL forward and 100 pmol/µL reverse primers (c-Myc and β-actin, respectively), QIAGEN OneStep RT-PCR Enzyme Mix, 40 U/mL RNase inhibitor (Promega), Digoxigenin-11-dUTP, and 2% bovine serum albumin (BSA; Sigma, USA) was prepared. The thermal cycling conditions were: 1 cycle at 95℃ for 5 minutes followed by 30 cycles for 30 seconds each at 94℃ (DNA denaturation), 30 seconds at 58℃ (primer annealing), 1 minute at 72℃ (primer extension), and 1 cycle of 8 minutes at 72℃ (final extension). After RT-PCR, the *in situ* frame was gently removed and the slides were flooded with PBS for 5 minutes, before being twice washed with PBS. Before detection of the PCR products, endogenous peroxidase in the breast tissue was blocked by incubation in 6% hydrogen peroxide in absolute methanol for 30 minutes in a dark humid chamber, followed by rinsing with PBS in Tween 20 for 2 minutes. Next,slides were treated with goat serum (0.05%; Jackson ImmunoResearch Laboratories, Inc., USA) for 15 minutes in a humid chamber. The slides were flooded with PBS in Tween 20 for 2 minutes and then washed for 10 minutes with PBS in Tween 20. Digoxigenin-11-2’-deoxy-uridine-5’-triphosphate- (Roche Diagnostics Ltd., Basel, Switzerland) labelled PCR products were detected by incubation with 60 µL anti-digoxigenin-POD (150 U/mL; Roche Diagnostics Ltd., Basel, Switzerland) diluted 1:80 in 100 mM Tris-HCl, 150 mM NaCl, pH = 7.5 for 30 minutes at room temperature in a humid chamber. The slides were washed with PBS in Tween 20 and then in deionised water. Sections were developed for 10-20 minutes at room temperature with 100 µL DAB chromogen (Millipore,USA). The slides were then kept in the dark and monitored at intervals until color development (brown color) in a dark humid chamber. The slides were flooded with PBS in Tween 20 and then rinsed in distilled water, before being counterstained with hematoxylin for 4 minutes which stains negative cells blue, then dehydrated in ascending concentrations of ethanol (75%, 95%, 100% I and 100% II) followed by xylene I and xyelene II for 5 minutes, each.

### Scoring system

The reaction intensity was variable, therefore a scoring system was used to score as follows: 0 (no signal); 1+ (mild intense); 2+ (moderate intense) and 3+ (strong intense). The distribution of reactivity within the target cell population on the mRNA reaction was recorded as follows: 0 (no positive cells), 1+ (1%-25% positive cells); 2+ (26%-50% positive cells); 3+ (51%-75% positive cells) and 4+ (75%-100% positive cells). Five random optical fields of each slide were scored.

### Statistical analysis

Data was expressed as mean ± standard deviation. Analysis of variance (ANOVA) was used to compare the means. A post hoc analysis was used to compare groups. P<0.05 was considered as statistically significant.

## Results

It was determined that a temperature of 52.8℃ produced the most intense, specific band on the gel for β-actin and c-Myc primers to be annealed to the target.

### Scoring of in situ RT-PCR

**Fig 1:**
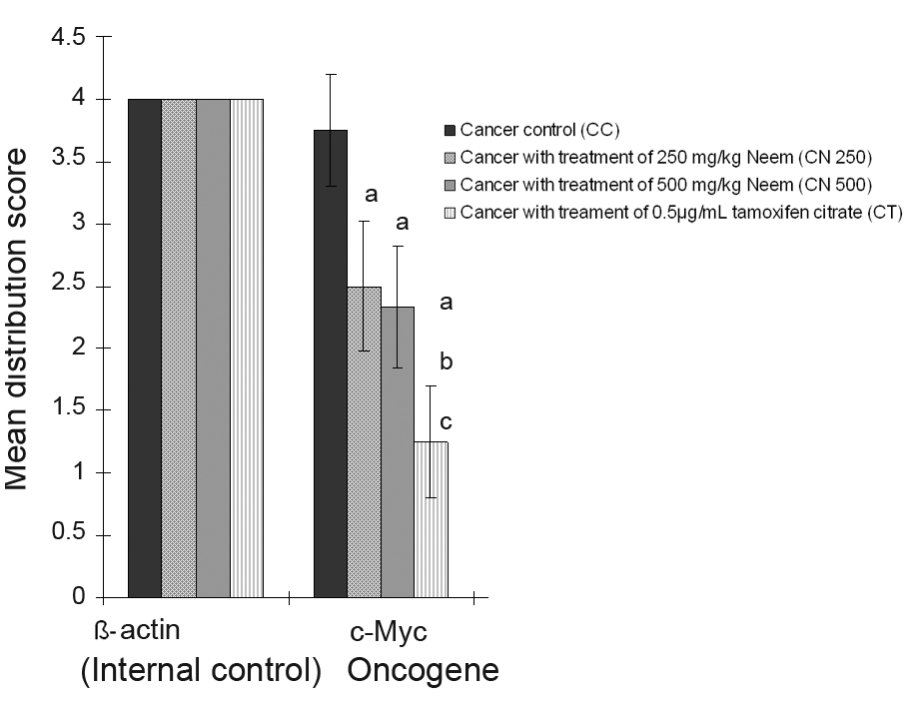
Mean distribution score of breast cancer oncogene (c-Myc) signal in 4T1 breast cancer mouse model under the effect of neem.Data are expressed as mean ± standard deviation. a = Significant difference with CC group at p<0.05. b = Significant difference with C250 group at p<0.05. c = Significant difference with C500 group at p<0.05.

**Fig 2:**
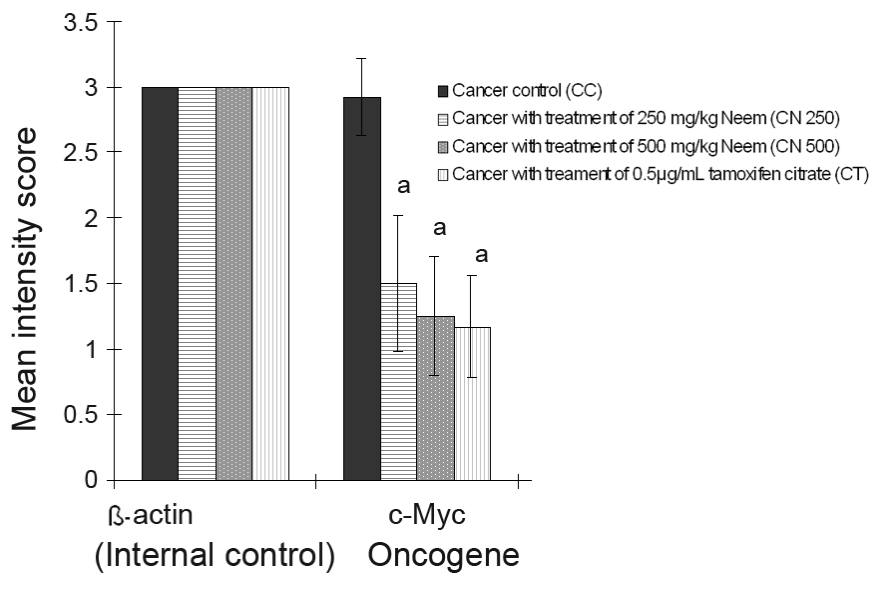
Mean intensity score of breast cancer oncogene (c-Myc) signal in 4T1 breast cancer mouse model under effect of neem. Data are expressed as mean ± standard deviation. a = Significant difference with CC group at p<0.05.

Figures 1 and 2 show the expression of the distribution and intensity of positive cells for β-actin and c-Myc mRNA by breast tumour cells of 4T1 mice using in situ RT-PCR with digoxigenin-11-dUTP.

### Controls for in situ RT-PCR

**Fig 3:**
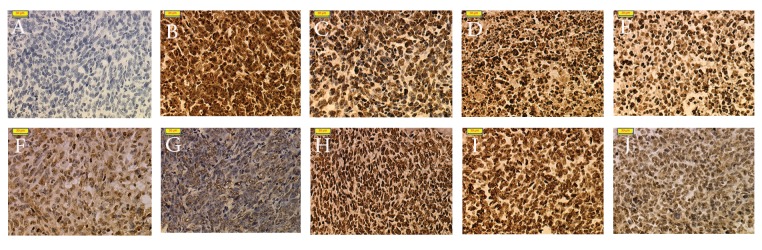
A. The negative control in situ RT-PCR with omission of primer showed no positive signal. B. The positive control in situ RT-PCR with no DNase treatment. The brownish color of the nucleus is very strong and intense. C. In situ RT-PCR detection of β-actin mRNA in breast tumour tissue of the CC group. A strong brown staining was observed in positive cells. D. In situ RT-PCR detection of β-actin mRNA in breast tumour tissue of the C250 group. A strong brown staining was observed in positive cells. E. In situ RT-PCR detection of β-actin mRNA in breast tumour tissue of the C500 group. A strong brown staining was observed in positive cells. F. In situ RT-PCR detection of β-actin mRNA in breast tumour tissue of the CT group. A strong brown staining was observed in positive cells. G. In situ RT-PCR detection of c-Myc mRNA in breast tumour tissue of the CC group. A strong brown staining was observed in positive cells. H. In situ RT-PCR detection of c-Myc mRNA in breast tumour tissue of the C250 group. A mild brown staining was observed in positive cells. I. In situ RT-PCR detection of c-Myc mRNA in breast tumour tissue of C500 group. A mild brown staining was observed in positive cells. J. In situ RT-PCR detection of c-Myc mRNA in breast tumour tissue of CT group. A mild brown staining was observed in positive cells. Magnification: x 200 for all slides.

For the negative control slide of *in situ* RT-PCR, we treated cells with DNase without the primers (Fig 3A). The negative control showed no positive signal, only hematoxylin as a nucleus background staining. Cells that were only pretreated with proteinase K and no DNase were considered the positive control. Fig 3B shows a very strong, intense brown color signal for the nuclei in the breast tumour cells.

### β-actin mRNA expression as internal control

The housekeeping gene β-actin showed similar distributions and intensity in both control and treated samples. The β-actin mRNA for the control and treated mice expressed at a strong intensity in more than 75% of positive cells. The β-actin mRNA signal was brown and intense in the nuclear region (Fig 3C-F). There were no significant differences in the distribution and intensity scores of β-actin in all experimental groups.

### Localization of c-Myc mRNA expression

*in situ* RT-PCR on breast cancer cells with c-Myc digoxigenin-labeled PCR product produced a positive signal which indicated expression of c-Myc mRNA in the breast cancer tissue. The results showed that c-Myc strongly expressed with the highest mean score of distribution (3.75 ± 0.45; p<0.05) and intensity (2.92 ± 0.29; p<0.05) in the breast cancer tissue of the CC group (Fig 3G), which was significantly higher compared to the other cancer treated groups. The mean distribution score of cancer treated with 250 and 500 mg/kg of neem and tamoxifen citrate were statistically similar. The positive cell distribution in the C250 group had a mean score of 2.50±0.52 and the positive cell distribution in the C500 had a mean score of 2.33±0.49 (Fig 3H,I). In the CT group, there was a less than 25% distribution of most of the stained cells (Fig 3J).

## Discussion

For thousand of years the evergreen neem tree, *Azadirachta indica* (A. Juss) has exhibited several properties that are medicinally useful. The plant and its preparations have been extensively reported to have insecticidal, pesticidal, and agrochemical properties. Additionally, its constituents are used in alternative (Ayurveda, Unani, homeopathy) and modern medicine to treat diverse infectious, metabolic, or cancerous conditions (12).

 There were many factors and parameters involved in the *in situ* RT-PCR methodology to avoid getting the false reactive results of the oncogene expression. The maintenance of an intact morphology

or the breast cancer tissues of the experimental mice regardless of the multiple steps of permea bilization and thermal cycling was crucial in this experiment. The negative control used with *in situ* RT-PCR showed no positive signal (Fig 3A) which indicated that the product detected was indeed cDNA and not a primer from contaminated genomic DNA. Whereas the positive control of the *in situ* RT-PCR (Fig 3B) showed a very strong, intense signal of nuclei in the 4T1 cells, which demonstrated the effectiveness of proteinase K digestion and success of the subsequent PCR steps. Over-digestion of proteinase K was manifested by the loss of the cell borders and cytoplasm (13). The entire process of *in situ* RT-PCR was well-controlled and the doubt of a false positive result was eliminated based on the results of the control. DNase digestion was performed prior to *in situ* RT-PCR to reduce nonspecific background amplification from genomic DNA (14). The highest concentration of DNase and the longest incubation did not eradicate all residual DNA (15). Consequently, the tissue was treated overnight with 20 U DNase, as the optimal recommended digestion time (8). If the tissue was adequately digested by protease, the genomic DNA would not be completely accessible to the DNase (16).

In addition, one potential cause of background can be eliminated by using digoxigenin, because it is not found in mammalian cells. Excellent results can be obtained over a broad range of concentrations of the labeled nucleotide (digoxigenin-11-dUTP) to the unlabeled nucleotide (dTTP) (17). The pre-mRNAs are present in the nucleus, whereas the spliced and modified mRNA is compartmentalized in specific regions of the cytoplasm or-in cases-in the nucleus (17). We used the β-actin housekeeping gene as an internal control of the expression level compared to the selected oncogenes for this experiment. Based on our results, the housekeeping gene β-actin showed similar distributions and intensity in both control and treated samples (Fig 3C-F) (17). These findings supported the initial assumption that the RNA was initially well preserved by the 10% buffered formalin, which proved that the levels of selected oncogene mRNA transcripts were not degraded because β-actin as an internal control showed a consistent level of expression among the different groups.

The variety in levels of expression of c-Myc oncogene under the effect of neem was judged confidently in reference to β-actin expression, without any doubt about issues of RNA degradation or false positive levels of expression in the c-Myc gene. The abnormal expression of c-Myc was reported in 32% of breast cancers (18). Between 1% and 94% (average = 15.5%) of breast cancer biopsies have a three-fold or greater c-Myc gene amplification (19), which has suggested its important role in genesis and/or progression of breast cancer (20).

Based on the results in figures 1 and 2, the c-Myc oncogene was dominant in the CC groups. Its expression was significantly higher compared to the C250, C500, and CT groups. c-Myc expression in the CT group was the lowest among the experimental groups, however the level of expression did not show significant difference between the three groups (C250, C500, CT).

Anti-estrogen tamoxifen (TAM) is effective in the treatment of estrogen receptor (ER)-positive as well as some ER-negative breast cancers. However, the precise mechanism of action of TAM, especially in estrogen-independent cells, remains un-clear (21). TAM-induced characteristic morphologic changes are consistent with apoptosis, including the compaction and margination of nuclear chromatin, the condensation of the cytoplasm, the convolution of the nuclear and cell outlines, and other typical biochemical changes such as internucleosomal DNA fragmentation (21).

Several studies have examined the role of the c-Myc proto-oncogene in cellular proliferation, transformation and mitogenesis, and programmed cell death (apoptosis). Blockage of the c-Myc expression with c-Myc antisense oligonucleotide confirmed that it is crucial for cell proliferation. Deregulation of c-Myc expression is associated with apoptotic cell death. Expression of c-Myc protein has been shown to be critical for the growth of both hormone dependent and hormone independent breast cancer cells (21).

This finding signified that under the treatment of neem, c-Myc mRNA expression was suppressed in cancerous mice. This has suggested that in a physiological situation, neem has the ability to reduce cell proliferation in response to the cancer in mice (22). In order to induce a tumour, c-Myc may need not only to promote cell proliferation, but also simultaneously inhibit its tendency for cell death, so as to increase cell numbers to form a tumour mass (23, 24). The suppressed expression of c-Myc in neem-treated cancer mice has shown a tendency towards suppressing the proliferation of cancerous cells and to induce apoptosis against 4T1 cancer cells.

A variety of chemotherapeutic drugs can initiate apoptosis in tumour cells, leading to the regression of a cancerous tumour (25). Previous work by our laboratory has demonstrated that neem leaf extract has high antioxidant activity, and cancerous mice treated with neem showed significantly higher values (p<0.05) in the means of body weight, apoptotic index, and apoptotic score compared to the control group. Furthermore, neem increased the mean survival time in the 4T1 breast cancer model (Unpublished data).

Subapriya et al. (26) demonstrated that pre-treatment of ethanolic leaf extract at a dose of 200 mg/kg significantly increased the antioxidant level in gastric cancer. Additionally, Manal et al. (27) have revealed that consumption of 5% of neem leaf aqueous extract resulted in increase of antioxidant enzymes to a level of complete inhibition of chemically induced hepatocarcinogenesis in rats. These studies would suggest that neem may contribute its greatest effect against the 4T1 breast cancer model not only via apoptosis induction but also by its antioxidant bioactivity. The overall results show the ethanolic neem leaf extract has a significant effect against breast cancer in the 4T1 mouse model. Further study is needed to understand the mechanism of neem leaf extract on oncogene expression.

## Conclusion

*in situ* RT-PCR showed that c-Myc oncogene expression was down regulated under stimuli of 500 mg/kg of ethanolic neem leaf extract.
